# Supplementary material for: Clay-Catalyzed Ozonation of Hydrotalcite-Extracted Lactic Acid Potential Application for Preventing Milk Fermentation Inhibition
Source: Molecules. 2022 Oct 1;27(19):6502. doi: 10.3390/molecules27196502 (PMC9572240; doi:10.3390/molecules27196502)
Supplement: Supplementary file 1 [file molecules-27-06502-s001.zip › molecules-1935215-supplementary.pdf]

Clay-catalyzed ozonation of hydrotalcite-extracted lactic acid
Potential application for preventing milk fermentation inhibition

Meriem El baktaoui ······· Abdelkrim Azzouz*

Supporting information

1. ***Catalyst characterization by X-ray diffraction***

XRD spectra of the main clay catalysts

X-ray diffractograms of the main clay materials used for this study (**Figure S1**) showed sharp and intense lines, providing evidence of their high crystallinity. The main XRD lines of Na^+-montmorillonite indicate that the clay is mainly composed of montmorillonite which is characterized by inter-reticular distances d_{101}= 11.40Å and d_{100} = 4.45Å. In NaMt, the inter-reticular distances of the d(101), d(101) and d(100) planes are of respectively 15.30, 4.45 and 4.2617Å. Bentonite purification into NaMt was found to remove volcanic ashes, dense silica phases and other amorphous impurities. The interlayer distance of NaMt is higher than for bentonite.

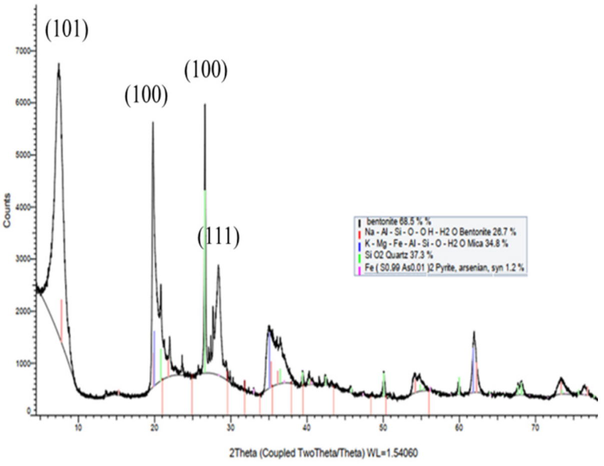

a

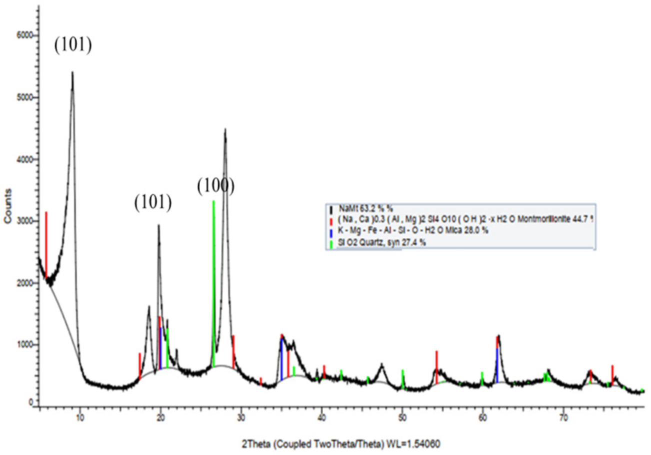

b

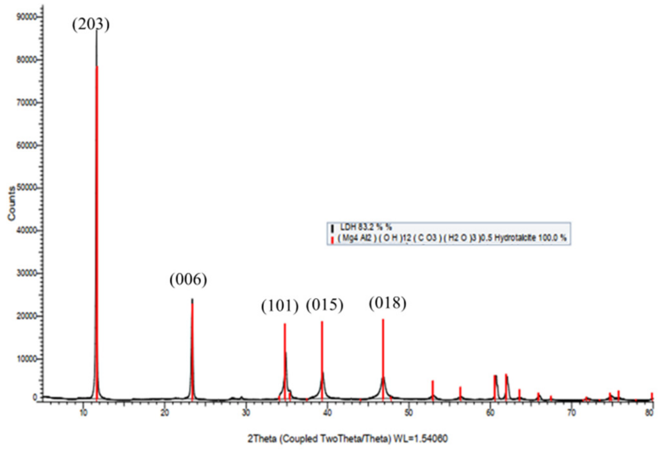

c

Figure S1. XRD spectra of bentonite (a), NaMt (b) and hydrotalcite (c).

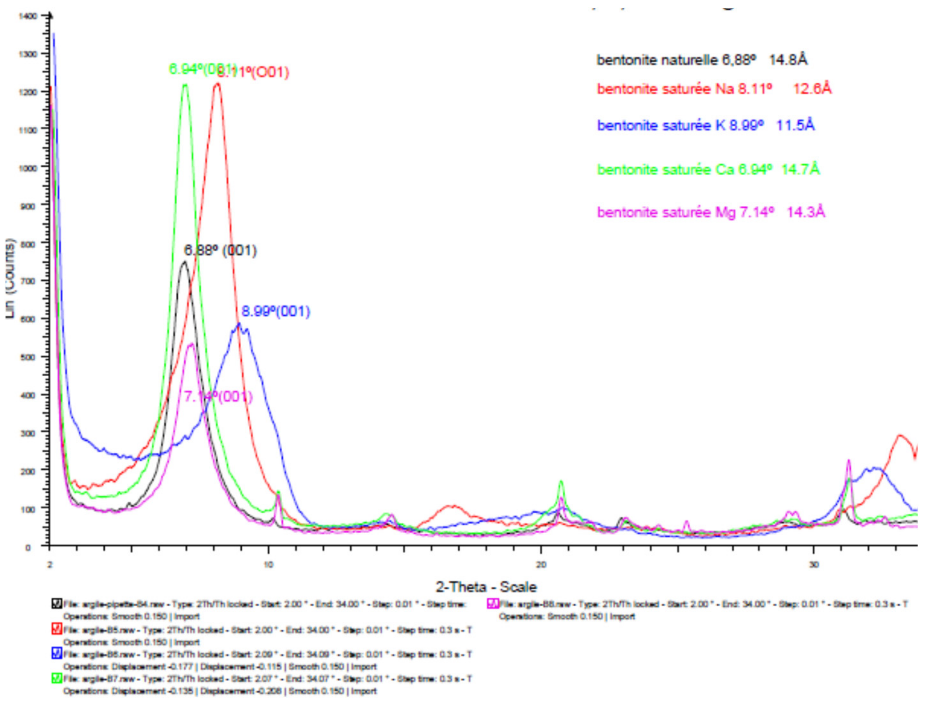

Figure S2. Effect of the exchangeable cation on the d_{001} basal spacing in bentonite and NaMt.

2. ***LA calibration plots and molar absorption coefficients***

Lactic acid (L (+) –LA, 88 % purity 88%, molecular weight 90.08 g.mol^{-1}) was supplied by Sigma aldrich. LA solutions with various concentrations (1, 2.5, 3, 4 and 5 g.L^{-1}) were prepared with nanopure water. Calibration curves were obtained by both UV-Vis spectrophotometry and high performance liquid chromatography coupled to UV dectection (HPLC-UV). This was achieved by plotting the optical density (absorbance) of LA UV-Vis bands and LA peak area obtained by HPLC-UV versus LA concentration (**Figure S1a**).

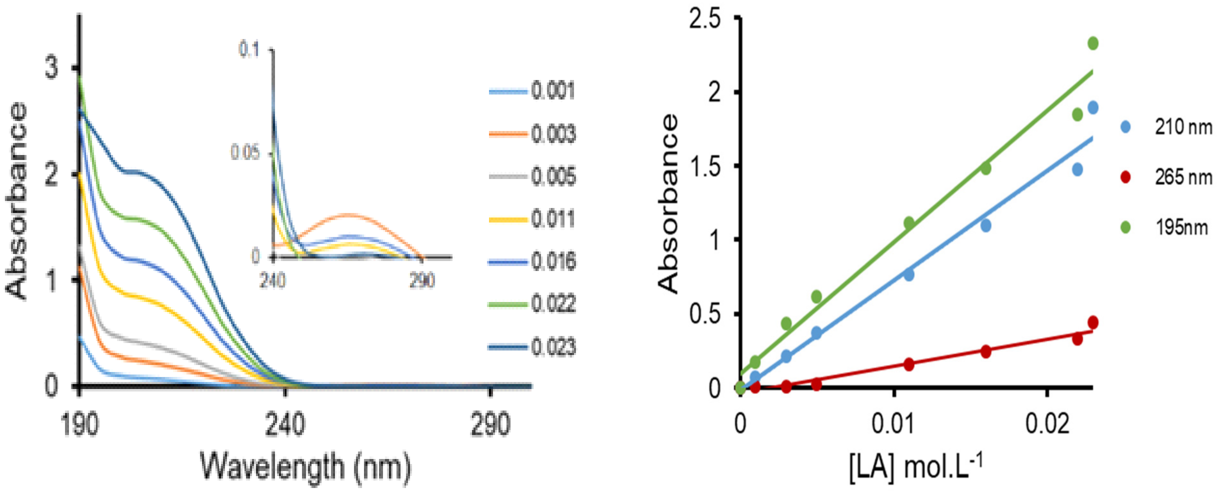

a *b*

Figure S3. UV-VIS spectra of lactic acid (***a***) and corresponding calibration plot (***b***) at room temperature. Various LA solutions (0.001-.0.0277 mol.L^{-1}) were prepared by successive dilutions from a stock LA solution (0.0277 mol.L^{-1}). Quartz cell: 1 cm.

Table S1. Molar absorption coefficient at different LA UV-Vis bands

Wavelength (nm)	Equation	R^2	Molar absorption coefficient ε (L.mol^{-1}.cm^{-1})
195	y = 0.4667x+0.0392	0.9943	177.986612
210	y = 0.7879x- 0.0124	0.9909	79.2686026
265	y = 0.4014x+0.0150	0.9702	42.2318293

3. ***Intensity increase in clay suspension in non-ozonized LA solution***

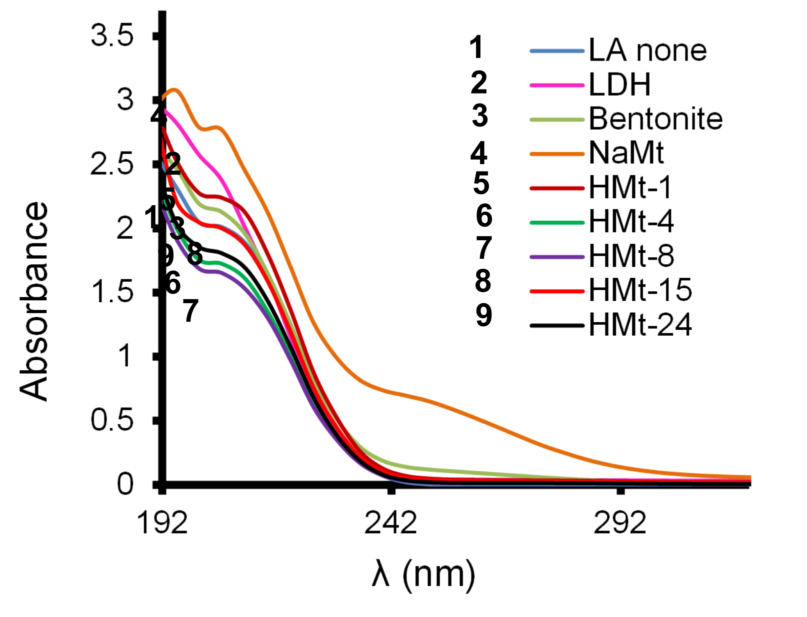

Figure S4. UV-Vis spectra of the supernatant of different clay suspensions (**a**) These spectra were recorded at T = 20 °C and intrinsic pH of the aqueous clay suspensions. Quartz cell = 1cm. Initial concentration 2.5 g. L^{-1} (27.75 mM). Contact time: 20 min.

4. ***Evolution in time of the intensity of the main UV-Vis bands during ozonation***

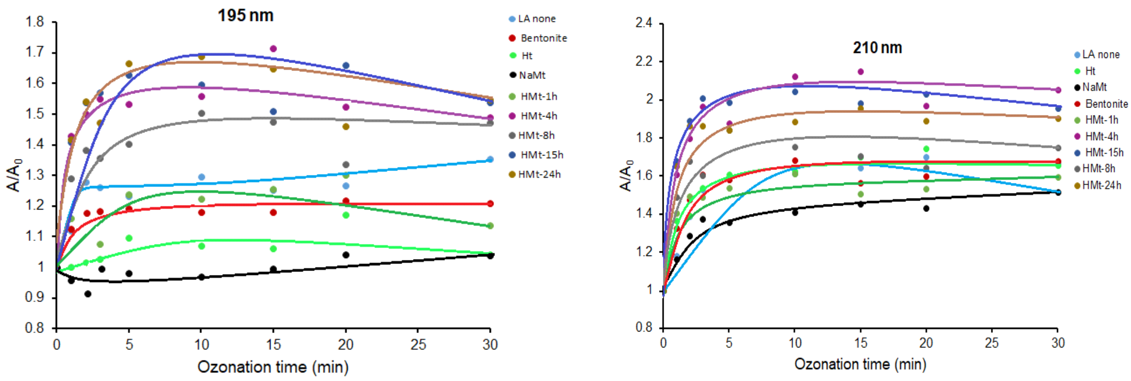

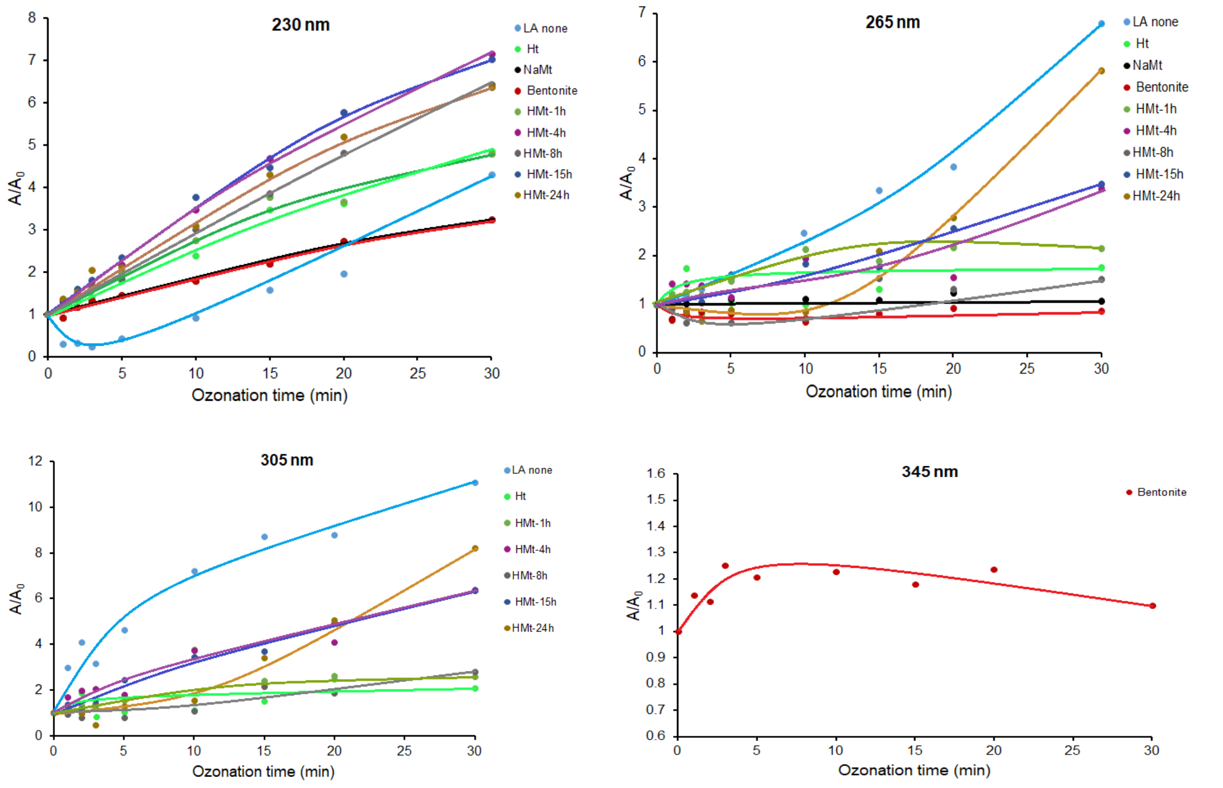

Figure S5. Evolution in time of the relative absorbance of the absorption bands of LA solution during the ozonation in the presence of the various catalysts.

5. *HPLC- UV calibration of LA concentration and reaction mixture analysis*

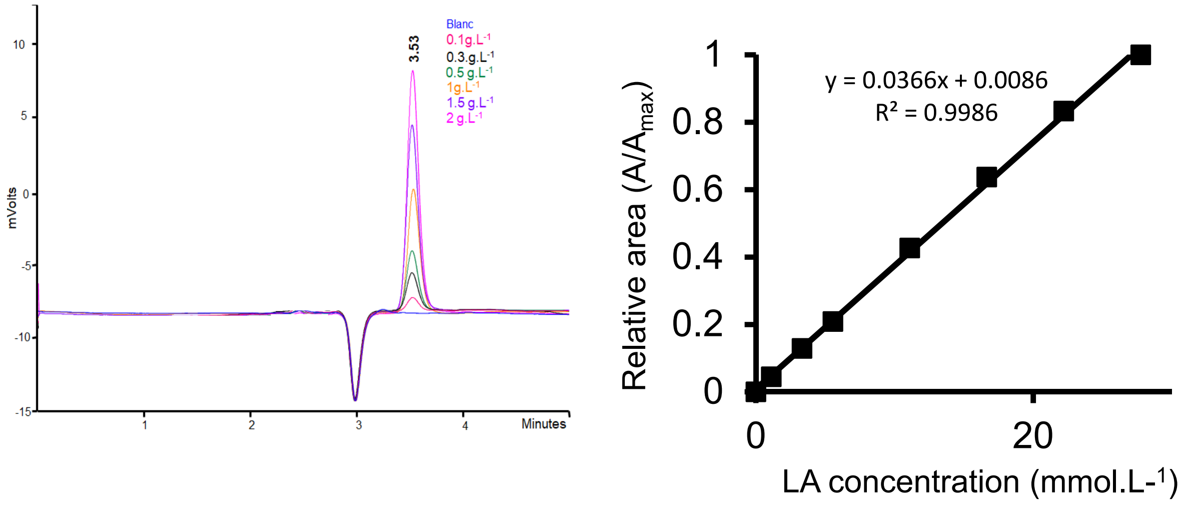

a *b*

Figure S6. HPLC-UV diagram of lactic acid at different concentrations (mmol.L^{-1}) as detected at 210 nm (***a***) and calibration curve of LA peak area (***b***). LA retention time : 3,53 min ; C18 column under a 1mL.min^{-1} throughput of an aqueous 0.06% solution of trifluoroacetic acid (TFA); T=20°C.

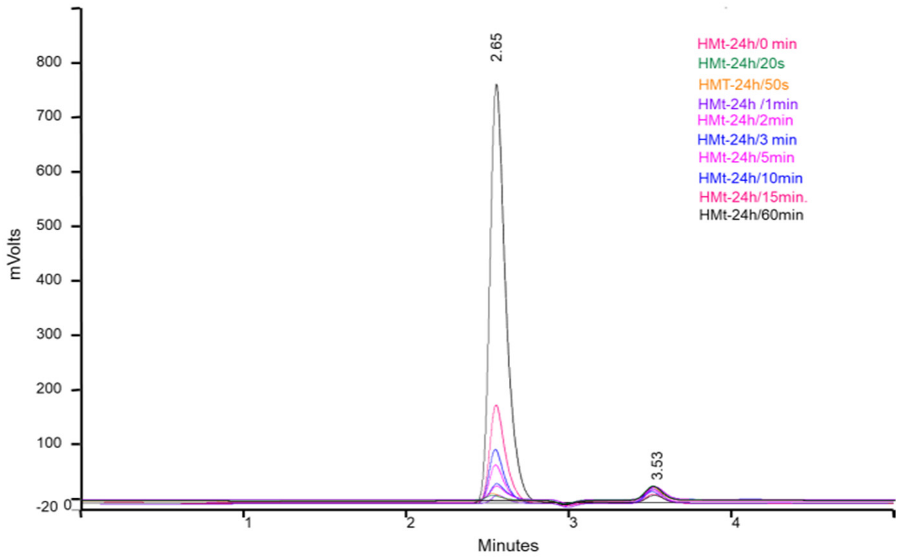

Figure S7. HPLC-UV chromatograms of LA solution after different ozonation times in the presence of HMt-24.

6. ***Product identification by LC-ToF-MS***

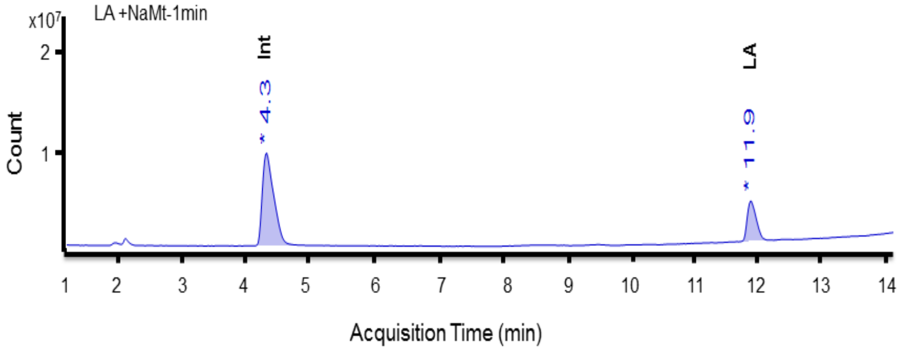

Figure S8. LC-Tof-MS chromatogram of ozonized LA solution in the presence of NaMt for 1 min. LC-ToF-MS in negative electrospray mode.

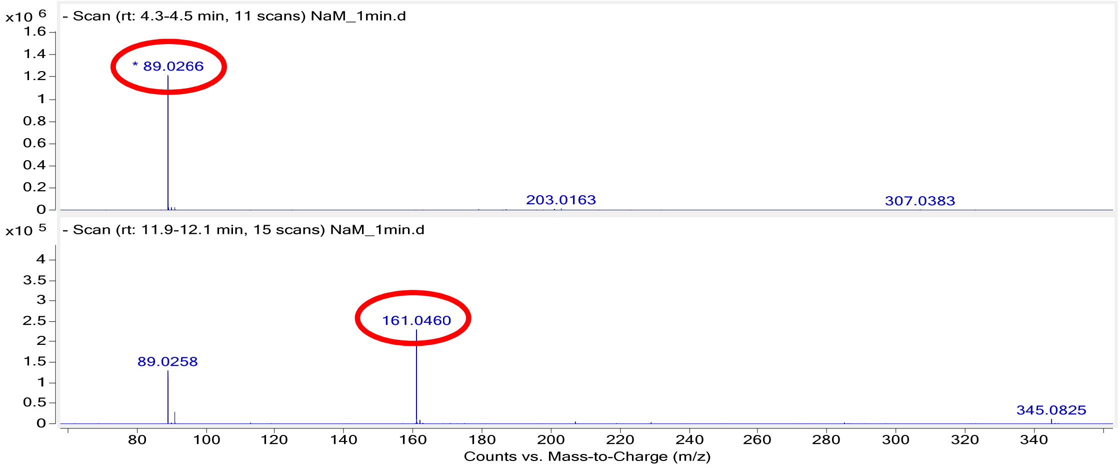

Figure S9. LC-ToF-MS diagram of the reaction mixture after 1 min NaMt-catalyzed ozonation.

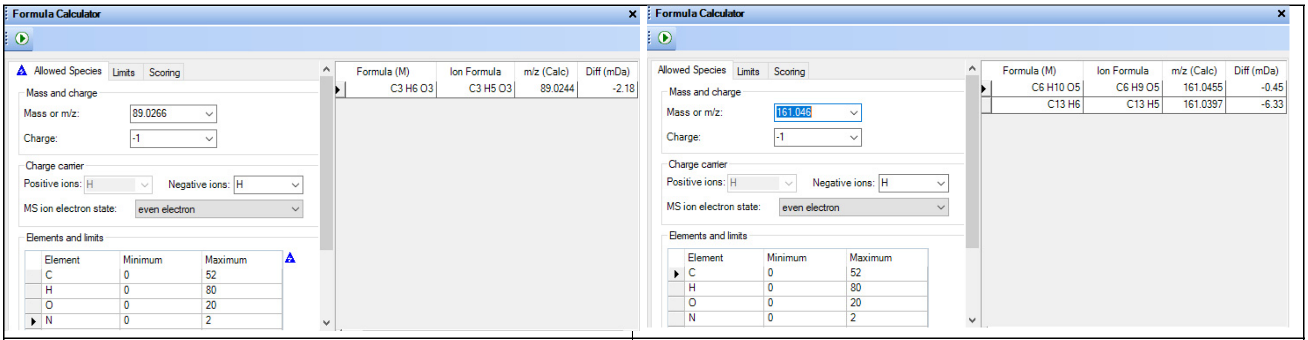

Identification of $C_3H_6O_3$ (Lactic acid) by m/z =89.0266	Identification of $C_6H_{10}O_5$ by m/z = 161.045

Figure S10. Identification of the main compounds detected by LC-ToF-MS.

7. *LA ozonation kinetics*

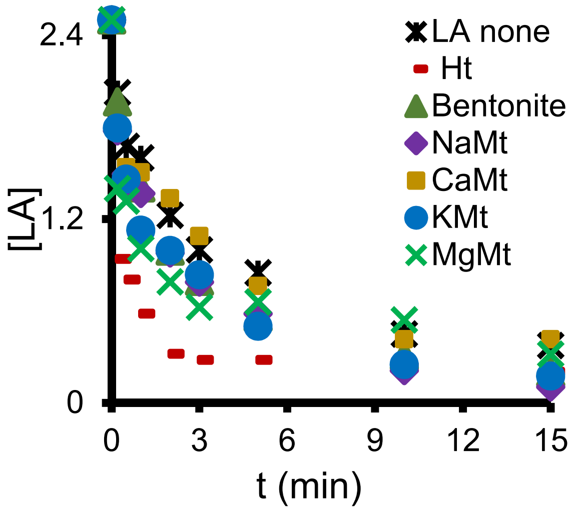

(A)

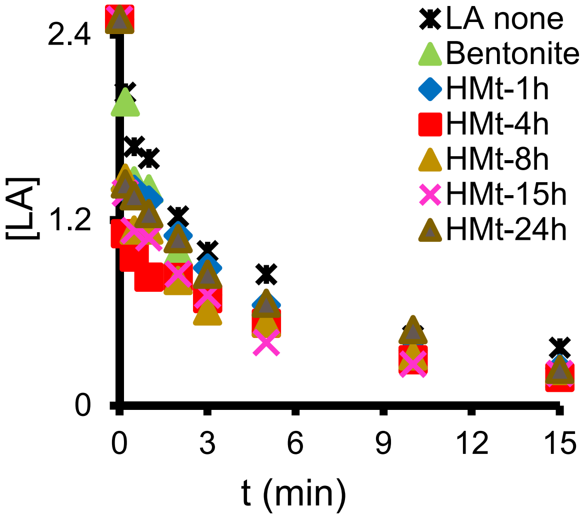

(B)

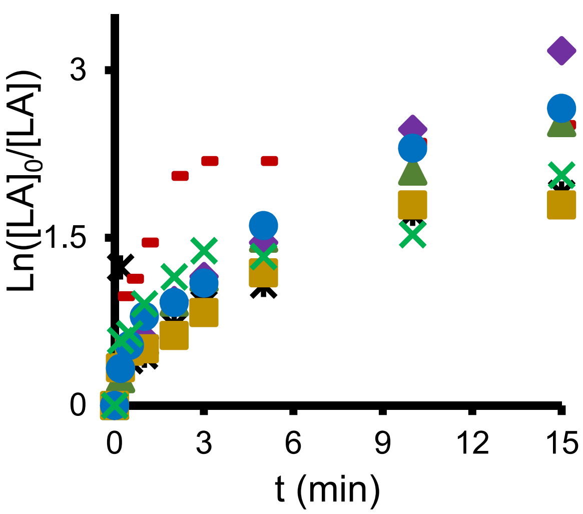

(C)

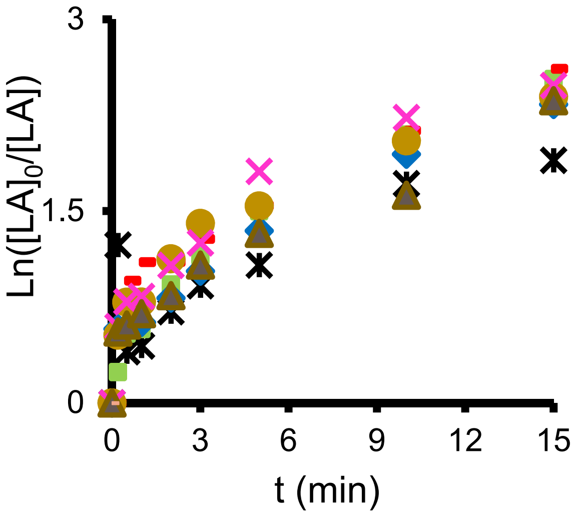

(D)

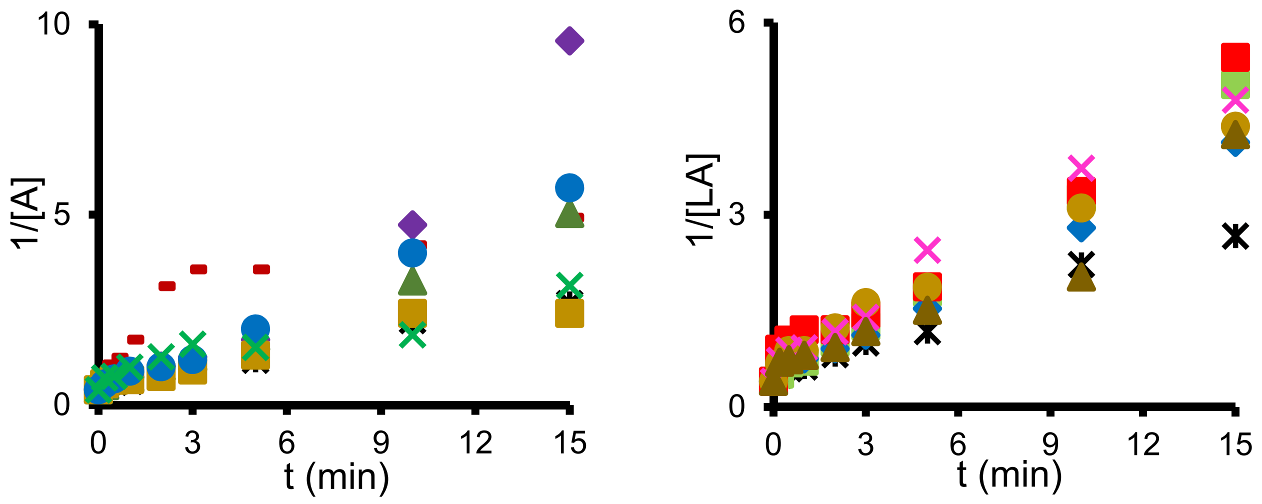

(E) **(F)**

Figure S11. Plots for Zero (**A, B**), 1st (**C,D**) and 2nd order (**E,F**) kinetical models applied to LA ozonation in presence of different clay minerals (**A,C,E**) and of activated bentonite (**B,D,F**) based on HPLC-UV measurements.

Table S2. Rate constant for Zero-order model of LA ozonation kinetics as assessed HPLC-UV.

Time range (min)	[0-3]		[0-5]		[5-15]	
	R^2	K (min^{-1})	R^2	K (min^{-1})	R^2	K (min^{-1})
None	0.8434	0.426255	0.7879	0.285507	0.865	0.0475
LDH	0.8932	0.341554	0.9186	0.278775	0.9231	0.04
Bentonite	0.7985	0.482283	0.7666	0.328128	0.9492	0.03605
NaMt	0.7932	0.455194	0.759	0.307775	0.9074	0.0478
CaMt	0.668	0.340697	0.7497	0.262983	0.75	0.03475
KMt	0.8516	0.3414	0.8674	0.261004	0.9119	0.0325
MgMt	0.6399	0.459412	0.5277	0.26208	0.9694	0.0341
HMt-1h	0.8512	0.244841	0.891	0.197577	0.9407	0.040875
HMt- 4h	0.485	0.268794	0.5194	0.181517	0.9611	0.035075
HMt- 8h	0.7862	0.371403	0.7202	0.238929	0.9518	0.030725
HMt- 15h	0.5682	0.394894	0.622	0.280437	0.9483	0.019975
HMt- 24h	0.852	0.259267	0.863	0.196299	0.9864	0.0428

Table S3. Rate constant for 1st order model of LA ozonation kinetics as assessed HPLC-UV.

Time range (min)	[0-3]		[0-5]		[5-15]	
	R^2	K (min^{-1})	R^2	K (min^{-1})	R^2	K (min^{-1})
None	0.1342	0.1359	0.2735	0.1245	0.9071	0.0818
LDH	0.9411	0.189	0.9749	0.1789	0.9521	0.0547
Bentonite	0.9125	0.346	0.9202	0.2755	0.9521	0.0547
NaMt	0.9233	0.3317	0.9119	0.2592	0.9912	0.1718
CaMt	0.7828	0.213	0.8943	0.1952	0.9988	0.0608
KMt	0.8547	0.2222	0.9471	0.2286	0.9922	0.1049
MgMt	0.8309	0.3808	0.6879	0.225	0.9922	0.1037
HMt-1h	0.8806	0.1435	0.9588	0.1558	0.9941	0.0989
HMt- 4h	0.7887	0.1992	0.8381	0.167	0.9926	0.107
HMt- 8h	0.9674	0.3029	0.9141	0.2211	0.8593	0.0853
HMt- 15h	0.7573	0.3088	0.8796	0.2823	0.9896	0.1037
HMt- 24h	0.9311	0.1595	0.973	0.1545	0.9685	0.1037

Table S4. Rate constant for 2nd order model of LA ozonation kinetics as assessed HPLC-UV.

Time range (min)	[0-3]		[0-5]		[5-15]	
	R^2	K (min^{-1})	R^2	K (min^{-1})	R^2	K (min^{-1})
None	0.9765	0.0928	0.9631	0.076	0.9685	0.0745
LDH	0.9683	0.0537	0.9839	0.0611	0.916	0.0383
Bentonite	0.9754	0.1354	0.9909	0.1332	0.9972	0.1622
NaMt	0.9804	0.1338	0.9899	0.1254	0.9824	0.3926
CaMt	0.8747	0.0698	0.9562	0.0792	0.75	0.0548
KMt	0.9567	0.1109	0.9699	0.1385	0.998	0.1854
MgMt	0.9566	0.1793	0.7944	0.1092	0.8951	0.0812
HMt-1h	0.9463	0.0819	0.9794	0.0909	0.9997	0.1298
HMt- 4h	0.7384	0.1174	0.8755	0.1098	0.9905	0.1792
HMt- 8h	0.968	0.171	0.9468	0.1349	0.9999	0.1257
HMt- 15h	0.8919	0.1389	0.9523	0.1768	0.9971	0.1171
HMt- 24h	0.9568	0.0902	0.9844	0.0895	0.8865	0.1372
